# Endothelial Dysfunction and Pre-Existing Cognitive Disorders in Stroke Patients

**DOI:** 10.3390/biom14060721

**Published:** 2024-06-18

**Authors:** Anne-Marie Mendyk-Bordet, Thavarak Ouk, Anne Muhr-Tailleux, Maud Pétrault, Emmanuelle Vallez, Patrick Gelé, Thibaut Dondaine, Julien Labreuche, Dominique Deplanque, Régis Bordet

**Affiliations:** 1 Univ. Lille, CHU Lille, Inserm, Lille Neuroscience and Cognition, F-59000 Lille, France; 2 Univ. Lille, CHU Lille, Inserm, Institut Pasteur de Lille, Nuclear Receptor, Metabolic and Cardiovascular Diseases, F-59000 Lille, France; anne.tailleux@univ-lille.fr (A.M.-T.); emmanuelle.vallez@pasteur-lille.fr (E.V.); 3 Univ. Lille, CHU Lille, Inserm, Biostatistic Platform, F-59000 Lille, France; 4 Univ. Lille, CHU Lille, Inserm, Department of Medical Pharmacology, F-59000 Lille, France

**Keywords:** Stroke, cognitive disorder, endothelial dysfunction, inflammation

## Abstract

Background: The origin of pre-existing cognitive impairment in stroke patients remains controversial, with a vascular or a degenerative hypothesis. Objective: To determine whether endothelial dysfunction is associated with pre-existing cognitive problems, lesion load and biological anomalies in stroke patients. Methods: Patients originated from the prospective STROKDEM study. The baseline cognitive state, assessed using the IQ-CODE, and risk factors for stroke were recorded at inclusion. Patients with an IQ-CODE score >64 were excluded. Endothelial function was determined 72 h after stroke symptom onset by non-invasive digital measurement of endothelium-dependent flow-mediated dilation and calculation of the reactive hyperemia index (RHI). RHI ≤ 1.67 indicated endothelial dysfunction. Different biomarkers of endothelial dysfunction were analysed in blood or plasma. All patients underwent MRI 72 h after stroke symptom onset. Results: A total of 86 patients were included (52 males; mean age 63.5 ± 11.5 years). Patients with abnormal RHI have hypertension or antihypertensive treatment more often. The baseline IQ-CODE was abnormal in 33 (38.4%) patients, indicating a pre-existing cognitive problem. Baseline IQ-CODE > 48 was observed in 15 patients (28.3%) with normal RHI and in 18 patients (54.6%) with abnormal RHI (*p* = 0.016). The RHI median was significantly lower in patients with abnormal IQ-CODE. Abnormal RHI was associated with a significantly higher median FAZEKAS score (2.5 vs. 2; *p* = 0.008), a significantly higher frequency of periventricular lesions (*p* = 0.015), more white matter lesions (*p* = 0.007) and a significantly higher cerebral atrophy score (*p* < 0.001) on MRI. Vascular biomarkers significantly associated with abnormal RHI were MCP-1 (*p* = 0.009), MIP_1a (*p* = 0.042), and homocysteinemia (*p* < 0.05). Conclusions: A vascular mechanism may be responsible for cognitive problems pre-existing stroke. The measurement of endothelial dysfunction after stroke could become an important element of follow-up, providing an indication of the functional and cognitive prognosis of stroke patients.

## 1. Introduction

Pre-existing cognitive impairment is a well-known risk factor for the occurrence of a post-stroke cognitive disorder, existing in over 10% of stroke patients [1]. Nevertheless, the origin of pre-existing cognitive disorders in stroke patients remains largely controversial. One hypothesis was that stroke could occur in patient with pre-existing Alzheimer’s disease, accelerating the evolution of cognitive impairment after stroke [2]. This hypothesis could be supported by the observation that post-stroke cognitive disorders could be associated with a degenerative process with amyloid deposition [3,4]. Nevertheless, these changes in amyloid metabolism could be a merely transient phenomenon [5]. Another hypothesis could be a vascular origin of the pre-existing cognitive disorders [6,7]. Indeed, stroke is associated with several vascular risk factors that are related to cognitive impairment as well as to vascular brain lesions that are involved in the occurrence of cognitive impairment [8]. Moreover, these vascular risk factors and brain lesions are associated with endothelial dysfunction that can contribute to cognitive impairment [9].

Endothelial dysfunction could be a marker supporting the vascular origin of pre-existing cognitive impairment in stroke patients. Endothelial dysfunction occurs as a result of oxidative stress [10,11] and leukocyte-endothelium interactions resulting from polymorphonuclear neutrophil activation [12], the induction of adhesion proteins (ICAM-1, VCAM-1) and the liberation of metalloproteases [9,13,14], which are molecular pathways involved in both stroke and dementia. Post-ischemic endothelial dysfunction contributes to more widespread tissue lesions, and its prevention leads to protection [8,13,14,15,16]. Clinical studies have demonstrated a difference in endothelial dysfunction depending on the stroke subtype [17] and vascular instability, which persists over the first few days post-stroke, increasing the risk of further vascular events [18].

We investigated whether pre-existing cognitive problems are associated with endothelial dysfunction in stroke patients and whether there is relationship with vascular risk factors, silent brain lesion and endothelium-related biomarkers.

## 2. Methods

### 2.1. Study Population

The study population was taken from an ongoing prospective, hospital-based, longitudinal cohort study set up to identify predictors for post-stroke dementia (STROKDEM study declared in clinicaltrial.org with the reference NCT01330160) [19,20]. The patients were included consecutively in the present study in the absence of the impossibility to perform measurements (conscious disturbance, lack of compliance); Details of the STROKDEM study design have been published previously [19,20]. In brief, adult patients admitted to the Lille University Hospital neurovascular intensive care unit (NICU) with acute ischemic stroke, dating from less than 72 h and defined by a focal neurological deficit in combination with a corresponding infarct on brain magnetic resonance imaging (MRI), were included. Patients were excluded if they had the following: a total score of >64 in the short version of the Informant Questionnaire on Cognitive Decline in the Elderly (IQ-CODE) [21], known CNS diseases other than stroke, a disease that may interfere with follow-up such as end-stage malignancy, or an inability to understand French.

### 2.2. Data Collection

For this study on endothelial dysfunction and pre-existing cognitive problems before stroke, the following data were recorded: complete medical history, risk factors for stroke and baseline cognitive state assessed using the IQ-CODE. The IQ-CODE uses the informant’s knowledge of the patient’s previous and current cognitive function. It measures 16 situations in which a person has to use their memory or intelligence. A cut-off value of 48 and above (mean score of 3 per item) was used to define cognitive impairment [22].

The following factors were measured 72 h after stroke: clinical state using the National Institute of Health Stroke Scale (NIHSS), endothelial function, magnetic resonance imaging (MRI) features and plasma biomarkers for endothelial damage.

### 2.3. Standard Protocol Approvals, Registrations and Patient Consent

The STROKDEM study was conducted according to the Declaration of Helsinki and was approved by the local ethics committee [20]. Written informed consent was obtained from all patients or their respondents prior to participation. Approval for surrogate consent was obtained to minimize recruitment bias.

### 2.4. Measurement of Endothelial Function

Endothelial function was assessed by the non-invasive measurement of endothelium-dependent flow-mediated dilation using an automated system (EndoPAT; Itamar Medical, Caesarea, Israel) [23]. This system is a plethysmograph capable of recording variations in blood volume at each pulse. Endothelial function was evaluated by measuring blood flow amplitude using digital sensors, first at rest and then after reactive hyperemia. Sensors were placed on the right and left index finger, and blood flow was measured continuously. A 5 min occlusion of the brachial artery (using a blood pressure cuff inflated to a pressure of 200 mmHg) was carried out on one of the two arms, and blood flow to the finger ceased temporarily. The cuff was then released, and an increase in blood flow to the finger was observed compared to the contralateral side. Measurement of this blood flow indicates how well the arteries dilate or endothelium-dependent relaxation.

A comparison of the blood flow to the ipsilateral and contralateral sides enables the calculation of the reactive hyperemia index (RHI), which is modified in cases of endothelial dysfunction. Patients were divided in two groups related to RHI: patients with a low RHI score ≤1.67 indicating endothelial dysfunction and patients with an RHI score ≥1.68 indicating normal function [24,25].

### 2.5. Measurement of Biomarkers of Endothelial Function

The number of polynuclear neutrophils was determined from blood counts. Homo-cysteine was measured by HPLC-mass spectrometry. Different biomarkers were analysed in blood or plasma samples: markers of endothelial activation (adhesion proteins, endo-thelial microparticles); markers of activation of polynuclear neutrophils (myeloperoxidase, neutrophil elastase, lactoferrin); markers of inflammation (cytokines, growth factor, metalloproteinases); markers of haemostasis (tissue factor, plasminogen, PAI-1, plas-min-antiplasmin complex). These measurements were carried out using Luminex^®^ commercially available kits (Bio-Plex System^®^, Bio-Rad Laboratories, Inc., Hercules, CA, USA) for the fluorescent multiplexing detection of plasma biomarkers or using classical commercial Enzyme Linked ImmunoSorbent Assays (ELISA). The Luminex^®^ technique is an advanced multiplexing technology used for the simultaneous detection and quantification of multiple biological targets, such as protein or peptides, in a single sample, combining fluorescent bead-based immunoassays with flow cytometry.

### 2.6. Evaluation by Imaging

All patients underwent MRI 72 h after stroke onset, and different parameters were determined, according to the recommendations of De Guio et al. [26]: ischemic or haemorrhagic lesions (size, location), lacunar lesions, cerebral or periventricular atrophy, and white matter changes. White matter lesions were scored using the FAZEKAS scale [27,28].

### 2.7. Statistical Analysis

Continuous variables are expressed as a mean (±standard deviation (SD)) in case of normal distribution or as a median (interquartile range (IQR)) otherwise. Normality was examined using histograms and the Shapiro–Wilk test. Ordinal (semi-quantitative scores) and non-ordinal categorical variables are expressed as numbers (percentage); the semi-quantitative FAZEKAS score ranging from 0 to 6 was also described using a median (IQR). For all biological parameters, the limit of detection was used for statistical analyses, and all comparisons were done using non-parametric tests (regarding the skewed distributions with a high rate of undetectable values for several variables).

Patients were categorized into two groups according to an abnormal RHI defined by a value ≤ 1.67. Bivariate comparisons between the two groups were made using Student’s *t* test for age, using Mann–Whitney U tests for vascular biomarkers and ordinal variables, and using a Chi-square test (or Fisher’s exact test when the expected cell frequency was <5) for categorical variables. A pre-specified adjustment for age was done for comparisons in clinical and biological findings, using binary or ordinal logistic regression models for clinical findings and using non-parametric analysis of covariance (ANCOVA) for biological findings.

We did not adjust for multiple comparisons given the exploratory nature of this study, and the present results should be considered as hypothesis-generating. Statistical testing was conducted at a two-tailed α level of 0.05. Data were analysed using the SAS software package, release 9.4 (SAS Institute, Cary, NC, USA).

## 3. Results

A total of 86 patients were included in the study (52 men and 34 women; mean age 63.5 ± 11.5 years (range 29–85)) (Table 1). Abnormal RHI (≤1.67) was detected in 33 patients (38.4%, 95%CI, 28.0% to 48.7%). As shown in Table 1, patients with abnormal RHI were older and had hypertension more frequently than patients with normal RHI. There is no significant difference between the two groups for the main stroke aetiologies (atherosclerosis: 12% vs. 8%; cardio-embolism: 21.5% vs. 19%; lacuna: 24% vs. 12%; percentage in abnormal RHI group vs. percentage in normal RHI group).

### 3.1. Endothelial Function and Pre-Existing Cognitive Disorders

Overall, 33 (38.4) patients were classified with cognitive impairment at baseline (IQ-code > 48). In the univariate analysis, baseline cognitive impairment was significantly more frequent in patients with abnormal RHI than in those with normal RHI (54.6% vs. 28.3%, *p* = 0.016). However, after adjustment for age, these differences did not reach the significance level, with an adjusted OR associated with an abnormal RHI of 2.42 (95%CI, 0.89 to 6.55; *p* = 0.082).

Regarding the vascular abnormalities at the 72 h MRI assessment, abnormal RHI was significantly associated with a higher FAZEKAS score (including periventricular lesions or white matter lesions) and atrophy score (Table 2). However, after adjustment for age, only the association of the abnormal RHI and atrophy score was significant (*p* = 0.045); the adjusted OR per 1-point improvement was 0.59 (95%CI, 0.24 to 1.43) for the FAZEKAS score and 0.31 (95%CI, 0.13 to 1.07) for the atrophy score.

### 3.2. Endothelial Function and Endothelium-Related Biomarkers

Table 3 shows a comparison of the distribution of vascular biomarkers of endothelium function according to abnormal or normal RHI. In the univariate analysis, compared to patients with normal RHI, patients with abnormal RHI had higher values in MCP-1 (*p* = 0.009), MIP_1a (*p* = 0.042), MIP_1b (*p* = 0.064), and VCAM-1 (*p* = 0.050), and lower values in S100B (*p* = 0.072). In the age-adjusted analyses, abnormal RHI remained associated with MCP-1 (*p* = 0.029) and with S100B (*p* = 0.022). Homocysteinemia was higher (*p* = 0.042) in patients with abnormal RHI (12.9 [10.6–15]) than in patients with normal RHI (11.3 [9.8–14.2]). However, when the age-adjusted *p*-values were corrected for multiple comparisons, none remained significant (*p* > 0.26).

## 4. Discussion

This study highlights a possible link between endothelial dysfunction and a pre-existing cognitive state before stroke and supports the hypothesis that in some patients a deterioration in the cognitive state may be vascular in origin. The weight of age in the role of endothelial dysfunction is not surprising in this study since it is the crucial and primary risk factor for both cognitive dysfunction and vascular aging, reinforcing the potential link between the two phenomena.

These observations support previous reports of a large group of conditions known as ‘mild cognitive impairment’ (MCI) [29,30] or ‘vascular dementia/vascular cognitive disorder’ (VaD/VCD) [31], which are known to be vascular in origin. It has been reported that 8–15% of older cognitively impaired subjects in Western countries and 22–35% in Japan have vascular dementia [31,32], with a prevalence in autopsy series of 0.03–58% [31,32]. In patients with VaD/VCD, multifocal and/or diffuse brain lesions ranging from lacunes to microinfarcts are found on neuropathological examination, often involving subcortical and strategically important regions of the brain (thalamus, fronto-basal, limbic system). Other damage, ranging from white matter lesions and hippocampal sclerosis to multi-infarct encephalopathy and diffuse post-ischemic lesions resulting from systemic, cardiac and local large and small vessel disease, are also observed [31,32]. In the heterogeneous condition MCI, amyloid plaques and neurofibrillary tau tangles have both been observed, and patients also have a cerebral vascular pathology, such as arteriosclerosis or cerebral amyloid angiopathy, which induces cerebral infarcts or hemorrhages of varying sizes and types, leading to further cognitive impairment [30].

Endothelial dysfunction has been associated with the existence of arterial hypertension in many studies [33,34,35]. In a review on the subject, Bernatova reported that endothelial dysfunction was not only a cause but was also an effect of hypertension [34]. Over half of our patients (54.7%) had hypertension, and a significantly higher proportion of patients with abnormal RHI were taking anti-hypertensive drugs when compared to those with normal RHI (69.7 vs. 45.3%; *p* = 0.027). Patients with endothelial dysfunction also had more white matter lesions, more periventricular lesions and a greater degree of cerebral atrophy. Endothelial dysfunction in these stroke patients is likely due to vascular inflammation, as shown by the abnormal serum levels of the chemotactic cytokines MCP-1 and MIP_1a. In a study of low-grade inflammation, endothelial dysfunction and cognitive function in older patients, Heringa et al. reported that low-grade inflammation and endothelial dysfunction contributed to reduced executive functioning and information processing speed in a population of older subjects [36]. Homocysteine plays a role in both endothelial dysfunction and cognitive impairment [37].

Our study also shows a close link between endothelial dysfunction, low-grade inflammation and the pre-existing cognitive state. IQ-CODE, assessed at baseline, was used to establish whether our patients had a pre-existing cognitive impairment before their stroke. This was found to be the case in over one-third (38.4%) of our patients. A comparison with MRI data revealed that endothelial dysfunction was associated with an increase in the number of ischemic or haemorrhagic microlesions, confirming the results of a recent study showing a link between endothelial dysfunction, lacunae and white matter lesions [17,38].

Cognitive disorders such as dementia are generally considered to be degenerative in origin [39]. However, a vascular origin has also been reported for some types of cognitive impairment [29,30,31,32]. It has been well established that vascular risk factors (diabetes, hypertension) are also risk factors for the occurrence of post-stroke cognitive disorders [40,41]. Post-stroke cognitive disorders are associated with many molecular mechanisms, including altered redox state, mitochondrial dysfunction, disruption of the blood–brain barrier, perivascular spacing, glymphatic system impairment, microglia activation and amyloid-β deposition in the parenchyma of the brain [42]. Our data support the hypothesis that in some patients, pre-existing cognitive problems before stroke are vascular in origin. This can explain the link between hypertension, as a well-known risk factor of stroke, and the occurrence of cognitive disorders [43]. Some molecular mechanisms, in particular vascular inflammation, could be similar between pre-existing and post-stroke cognitive impairment.

The present findings are derived from observational analyses that are subject to well-known limitations. The first is the potential for confounding by measured or unmeasured variables, which cannot be ruled out, even after adjustment. For example, patients with endothelial dysfunction are older, but this undoubtedly reflects the longer process of endothelial alteration required to impact cognitive function. The second limitation is that we could not exclude that some differences may have been overlooked due to the lack of adequate statistical power regarding our small study sample size. In a posterior power calculation, we calculated the smallest significant between-group difference (expressed as a standardized difference) that our study sample size allowed us to detect with 80% power. With 53 patients with normal RHI and 33 patients with abnormal RHI, we could detect a standardized difference of 0.63, interpreted as a medium-to-large effect. On the other hand, regarding the multiple testing issue, we caution that we could not exclude false positive findings. The third limitation is that endothelial dysfunction was only measured in periphery, while remaining cautious about making extrapolations in the cerebral circulation [43]. Nevertheless, endothelial dysfunction is a systemic phenomenon, and the association of hypertension with both endothelial dysfunction and risk of stroke or cognitive disorders suggests that a link may exist between peripheral and brain consequences, in particular in terms of prevention. For all these reasons, the present findings are only considered as hypothesis-generating and should be replicated in further studies, with a large stroke population size.

The results of this study highlight the possible contribution of a vascular mechanism to cognitive problems that pre-exist stroke. It could suggest that monitoring endothelial dysfunction post-stroke could become an important element of follow-up, providing an indication of the functional and cognitive prognosis of patients. It is important to study the effect of medical treatments of risk factors for endothelial dysfunction in more detail, using a larger study population.

## Figures and Tables

**Table 1:** Demographics characteristics and vascular risk factors overall and according to the normality of the reactive hyperemia index.

	Total	RHI		*p*
		Normal (>1.67)	Abnormal (≤1.67)	
No. (%) of patients	86	53 (61.6)	33 (38.4)	
Age (years), mean ± SD	63.5 ± 11.5	59.6 ± 10.8	69.7 ± 9.9	<0.0001
Gender, male, n (%)	52 (60.5)	33 (62.3)	19 (57.6)	0.67
Vascular risk factors, *n* (%)				
Arterial hypertension	47 (54.7)	24 (45.3)	23 (69.7)	0.027
Diabetes	15 (17.4)	8 (15.1)	7 (21.2)	0.47
Hypercholesterolemia	40 (46.5)	23 (43.4)	17 (51.5)	0.46
Smoking	19 (22.1)	10 (18.9)	9 (27.3)	0.36
Excessive alcohol	10 (11.6)	5 (9.4)	5 (15.1)	0.50
Physical activity	54 (62.8)	34 (64.2)	20 (60.6)	0.74
Treatments, *n* (%)				
Antihypertensive drugs	47 (54.7)	24 (45.3)	23 (69.7)	0.027
Oral hypoglycemics	11 (12.8)	6 (11.3)	5 (15.2)	0.74
Statins	28 (32.6)	14 (26.4)	14 (42.4)	0.12

Abbreviations: SD: standard deviation; RHI: reactive hyperemia index.

**Table 2:** Association of abnormal reactive hyperemia index with clinical findings at 72 h.

	RHI		
	Normal (>1.67) (*n* = 53)	Abnormal (≤1.67) (*n* = 33)	*p* */†
IQ-CODE > 48, *n* (%)	15 (28.3)	18 (54.6)	0.016/0.082
Total FAZEKAS score, median (IQR)	2.0 (1.0 to 3.0)	2.5 (2.0 to 3.5)	0.008/0.22
0	8 (15.7)	2 (6.2)	
1	11 (21.6)	2 (6.2)	
2	17 (33.3)	12 (37.5)	
3	10 (19.6)	8 (25)	
4	5 (9.8)	3 (9.4)	
Periventricular lesions, *n* (%)			
0	10 (19.6)	3 (9.4)	0.015/0.38
1	26 (51.0)	12 (37.5)	
2	14 (27.4)	12 (37.5)	
3	1 (2.0)	5 (15.6)	
White matter lesions, *n* (%)			
0	17 (33.3)	4 (12.5)	0.007/0.10
1	30 (58.8)	20 (62.5)	
2	4 (7.8)	6 (18.8)	
3	0 (0.0)	2 (6.3)	
Atrophy score, *n* (%)			
0	14 (27.5)	1 (3.1)	<0.001/0.045
1	28 (54.9)	16 (50.0)	
2	9 (17.6)	12 (37.5)	
3	0 (0.0)	3 (9.4)	
Previous lacunar infarct, *n* (%)	11 (21.2)	10 (31.3)	0.30/0.94
Perivascular atrophy, *n* (%)	5 (9.8)	8 (25.0)	0.064/0.41

* unadjusted *p*-values/† age-adjusted *p*-values. Abbreviations: IQCODE: Informant Questionnaire on Cognitive Decline in the Elderly; RHI: reactive hyperemia index.

**Table 3:** Association of abnormal reactive hyperemia index with vascular biomarkers of endothelial dysfunction.

	RHI		
	Normal (>1.67) (*n* = 53)	Abnormal (≤1.67) (*n* = 33)	*p* */†
SE_selectin, ng/mL	29.6 (10.2 to 37.9)	30.6 (19.6 to 38.4)	0.72/0.39
BDNF, pg/mL	4003 (2146 to 5466)	3814 (2301 to 5439)	0.96/0.93
ICAM-1, µg/mL	132,390 (86,536 to 189,955)	160,772 (117,946 to 192,783)	0.22/0.39
MPO, ng/mL	86,565 (62,791 to 178,239)	133,569 (62,791 to 336,719)	0.23/0.32
NCAM, ng/mL	360,421 (302,422 to 467,724)	367,789 (315,689 to 446,092)	0.80/0.61
VCAM-1, ng/mL	1,320,000 (956,802 to 1,710,000)	1,520,000 (1,300,000 to 1,700,000)	0.051/0.12
S100B, ng/mL	50 (27 to 125)	27 (27 to 80)	0.072/0.022
G-CSF, µg/mL	8.8 (4.9 to 19.7)	4.9 (4.9 to 19.7)	0.38/0.29
GM_CSF, µg/mL	1.6 (1.6 to 1.6)	1.6 (1.6 to 1.6)	0.41/0.39
MCP-1, pg/mL	3.0 (3.0 to 7.8)	14.7 (3.0 to 36.9)	0.009/0.029
MIP_1a, ng/mL	0.5 (0.1 to 1.1)	1.0 (0.1 to 1.5)	0.042/0.14
MIP_1b, ng/mL	43 (31 to 59)	53 (36 to 82)	0.064/0.23

Values are median (IQR). * unadjusted *p*-values/† age-adjusted *p*-values. Abbreviations: IQR = interquartile.

## Data Availability

The original contributions presented in the study are included in the article, further inquiries can be directed to the corresponding author.
